# Molecular Mechanism of Vine Tea Dihydromyricetin Extract on Alleviating Glucolipid Metabolism Disorder in *db/db* Mice: Based on Liver RNA-Seq and TLR4/MyD88/NF-κB Pathway

**DOI:** 10.3390/ijms26052169

**Published:** 2025-02-28

**Authors:** Xixin Zhou, Xin Liu, Yuhang Yi, Shiyun Chen, Yi Zhang, Wei Fan, Chenghao Lv, Si Qin

**Affiliations:** 1 College of Bioscience and Biotechnology, Hunan Agricultural University, Changsha 410128, China; zhouxixin@hunau.edu.cn (X.Z.); yu_xin@stu.hunau.edu.cn (X.L.); yiyuhang@stu.hunau.edu.cn (Y.Y.); zyi1219@163.com (Y.Z.); 2 College of Food Science and Technology, Hunan Agricultural University, Changsha 410128, China; shiyunchen0531@stu.hunau.edu.cn (S.C.); weifan@hunau.edu.cn (W.F.); 3 Xiangya School of Basic Medical Sciences, Central South University, Changsha 410013, China

**Keywords:** vine tea, dihydromyricetin, glucolipid metabolic disorder, RNA-seq, TLR4/MyD88/NF-κB pathway

## Abstract

The primary active compound in vine tea is dihydromyricetin (DMY), which has a longstanding history as a dietary supplement and traditional ethnic medicine. However, the precise molecular mechanism by which vine tea dihydromyricetin extract (VDMY) regulates glucolipid metabolic disorder remains unclear. In this study, we first assessed the effect of VDMY on various physiological parameters in *db/db* mice, followed by RNA sequencing (RNA-seq) to identify key signaling pathways affected by VDMY in liver tissues. We also examined the impact of VDMY on the liver’s TLR4/MyD88/NF-κB and FOXO1 pathways using Western blotting. Our results showed that VDMY significantly reduced fasting blood glucose (FBG), total cholesterol (TC), triglycerides (TGs), and low-density lipoprotein cholesterol (LDL-C), while increasing high-density lipoprotein cholesterol (HDL-C) levels. Additionally, VDMY enhanced the liver’s antioxidant capacity by upregulating superoxide dismutase (SOD), catalase (CAT), and glutathione (GSH), while lowering malondialdehyde (MDA), alanine aminotransferase (ALT), and aspartate aminotransferase (AST), thus alleviating liver damage. RNA-seq analysis further revealed that VDMY influenced multiple biological processes, including transcription, glycolysis, gluconeogenesis, and redox reactions, suggesting that its effects may be mediated through the TLR4/MyD88/NF-κB and FOXO1 pathways. Additionally, Western blot analysis revealed that VDMY effectively downregulated the expressions of TLR4, MyD88, NF-κB, and FOXO1 proteins in the liver of *db/db* mice, indicating that VDMY could target these pathways to intervene glucolipid metabolism dysfunction.

## 1. Introduction

With the continuous advancement of modernization, human lifestyles have undergone profound changes. These changes, while bringing convenience, have also made glycolipid metabolism disorder (GLMD) an increasingly prominent chronic disease. Its impact on the global healthcare system and clinical practice cannot be ignored [1]. GLMDs are defined as conditions that involve disturbances in the metabolism of glucose and lipids [2]. Multiple causative factors, ranging from genetic predisposition to complex environmental triggers to the deeper influence of individual psychological state and dietary patterns, together form the basis for the pathogenesis of these diseases. The pathological mechanisms that underlie GLMD include oxidative stress, inflammatory processes, insulin resistance, dyslipidemia, and alterations in gut microbiota composition [3]. Clinically, individuals with these disorders may exhibit symptoms such as hyperglycemia, dyslipidemia, and hypertension [4]. When glucose and lipid metabolism disorders occur, inflammatory factors and immune cells create a complex biochemical storm in key areas such as blood vessel walls, pancreatic islet tissue, liver parenchyma, and adipocytes. This microscopic level of inflammatory damage is not isolated, but rather occurs through a complex network of inflammatory mediators and signal transduction pathways, forming a multi-organ pathological mechanism that is interrelated and interdependent, thus exacerbating the progression and alleviation of the disease [5].

GLMD is often associated with insulin resistance, with chronic nutritional excess recognized as a prominent factor contributing to systemic insulin resistance. This sustained nutritional surplus enhances pro-inflammatory signals and activates inflammation signaling pathways in macrophages, particularly through the activation of c-Jun NH2-terminal kinase (JNK). This complex process of cellular activation triggers a series of cascading reactions, the most striking of which is the secretion of a variety of inflammatory factors, and of particular interest is the significant release of key inflammatory mediators, such as tumor necrosis factor-alpha (TNF-α) and interleukin-1beta (IL-1β), during this process [6]. The heightened activity of TNF-α stimulates lipolysis, which subsequently leads to elevated concentrations of free fatty acids [7]. Abnormal accumulation of free fatty acids disrupts the liver’s homeostatic balance. It also triggers the remodeling of the triglyceride (TG) metabolic pathway through a series of cascade reactions. This remodeling ultimately leads to significant changes in the lipid profile. Specifically, it causes an abnormal rise in low-density lipoprotein cholesterol (LDL-C) and a continuous decline in high-density lipoprotein cholesterol (HDL-C) levels [8]. In this complex metabolic storm, the overexpression of the inflammatory factors TNF-α and IL-1β undoubtedly plays a contributing role, constantly promoting the uncontrolled proliferation of reactive oxygen species (ROS). It is worth pondering that, as an unavoidable by-product of cellular metabolism, the uncontrolled accumulation of ROS implies the collapse of the body’s oxidative stress balance, and this imbalance is pathologically related to obesity and the various metabolic disorders it causes [9]. An elevation in ROS level is linked with a marked decrease in endogenous antioxidant molecules, such as superoxide dismutase (SOD), catalase (CAT), and glutathione peroxidase (GSH-Px). These reductions contribute to oxidative stress and may precipitate metabolic syndrome, including obesity and type 2 diabetes mellitus (T2DM), thereby creating a detrimental vicious cycle [10]. Meanwhile, ROS interact with specific structural domains of nuclear factor-kappa B (NF-κB) through a unique intermolecular mechanism, and this subtle but critical interaction triggers a significant change in the conformation of the NF-κB protein, which in turn has a profound effect on its intrinsic activity at the molecular level and ultimately leads to a substantial alteration of the function of this important transcription factor [11]. As an essential intracellular transcription factor, NF-κB plays an indispensable role in the regulation of a wide range of physiological processes, covering areas ranging from inflammatory responses to immune regulation to cell survival. It is interesting to consider that when the activity level of NF-κB is abnormally elevated, such changes often cause significant disruptions in the insulin signaling system, particularly through the inhibition of key signaling molecules, including Insulin Receptor Substrate 1 (IRS-1), which ultimately leads to insulin dysfunction [12]. Concurrently, lipopolysaccharides (LPSs) in the individual migrate from the enteric cavity into the circulatory system, which in turn triggers the synthesis of endogenous ethanol and short-chain fatty acids (SCFAs). This mechanism provokes an immune response in the liver, resulting in disruptions to glucose metabolism and related inflammatory injury. LPS possesses the ability to activate inflammatory receptors, such as Toll-like receptor 4 (TLR4) and NF-κB, thereby facilitating the release of inflammatory mediators by immune cells [13].

Vine tea is also known as “Mei” tea, which is classified within the *Ampelopsis grossedentata*. Its edible parts are young stems and leaves. Vine tea is a commonly used food and medicine health tea for Tujia, Zhuang, and other nationalities in Hunan, Hubei, Guizhou, and Jiangxi in China [14]. It is rich in bioactive compounds, including flavonoids, polysaccharides, alkaloids, and polyphenols, which can play various biological activities. The content of flavonoids in vine tea can reach about 45%, which is known as the “King of flavonoids”. The most important flavonoid in vine tea with the highest content is DMY, which has a mass fraction as high as 30% or more in dry stems and leaves of vine tea [15] According to Zhang et al., flavonoids in vine tea mainly include dihydromyricetin (DMY), myricetin, myricitrin, and myricetin-3-β-D-galactopyranoside [16]. Among the constituents, dihydromyricetin exhibits the highest concentration, with a mass fraction reaching up to 35% [17].

With the continuous deepening of scientific research, DMY, the active ingredient in Garcinia Cambogia, has shown remarkable pharmacological value. Recent studies have indicated that DMY found in vine tea exhibits a range of beneficial properties, including anti-inflammatory, antibacterial [18], anti-tumor [19], and antioxidant effects [20]. Furthermore, it has been shown that DMY is particularly effective in lowering blood lipid levels [21] and blood glucose [22], thereby protecting cardiovascular health [23]. DMY exhibited significant glucose regulation in T2DM mice; this natural compound also effectively elevated serum levels of adiponectin, and notably, it showed unique therapeutic potential in improving symptoms of insulin resistance [24]. In addition, DMY can regulate the critical transcription factor SREBP-1c, which is involved in lipid synthesis; diminish TG accumulation; and improve hepatic steatosis [25]. DMY has been shown to mitigate lipid accumulation associated with insulin resistance [26] and mitochondrial dysfunction [27]. It also restores the antioxidant enzyme system [28] and reduction levels of liver lipid peroxidation, as indicated by Thio barbituric acid reactive substances (TBARSs), thereby improving oxidative stress in the liver [29]. Furthermore, DMY enhanced insulin sensitivity and alleviated symptoms of metabolic syndrome by modulating the phosphorylation of insulin receptor substrate-1 at tyrosine 612 (Y612) in *db/db* mice [30]. Furthermore, DMY has the capacity to reverse alterations in glycolysis and the tricarboxylic acid (TCA) cycle in diabetic murine models. It can decrease the concentrations of fructose-1,6-bisphosphate, glucose-6-phosphate, fructose-6-phosphate, and other metabolites in the liver, thereby ameliorating carbohydrate metabolism disorders through the reduction of glycolytic intermediates [31]. Additionally, DMY inhibited gluconeogenesis via the Akt/FoxO1/PCK2 signaling pathway [32]. The molecular mechanisms through which it targets inflammation-related signaling pathways to regulate glucolipid metabolic disorder remain unclear.

This study obtained DMY from vine tea (VDMY) as the material to investigate the molecular mechanism of its regulation of glucose and lipid metabolism disorders by RNA-seq technology, as well as its effects on physicochemical indexes such as blood glucose, blood lipid, and oxidative stress in *db/db* mice. Furthermore, how DMY regulates the GLMD via the TLR 4/Myd88/NF-κB signaling pathway was explored by RNA-Seq and Western blot analysis.

## 2. Results

### 2.1. Effects of VDMY on Blood Biochemical Indicators in db/db Mice

**Figure 1:**
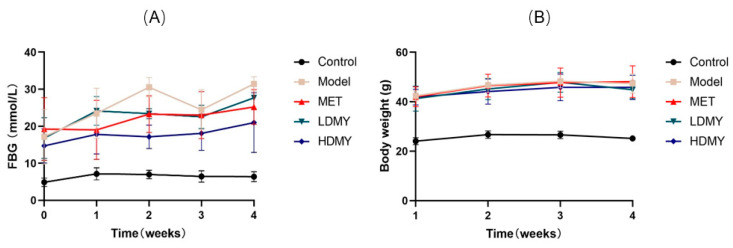
Effect of VDMY on blood glucose and body weight in *db/db* mice. (**A**) Fasting blood glucose (FBG) was detected in control, model, MET, LDMY, and HDMY groups, at the checkpoints of 0, 1, 2, 3, and 4 weeks. (**B**) Body weight was detected in control, model, MET, LDMY, and HDMY groups, at the checkpoints of 1, 2, 3, and 4 weeks. There is no significance between each group.

Firstly, a *db/db* mice experiment was performed to verify the anti-diabetic effect of VDMY at the in vivo level. As illustrated in Figure 1A, the initial blood glucose levels of all groups were similar. From the second week onwards, the blood glucose levels in the MET, LDMY, and HDMY groups began to decrease, and they remained lower than those of the model group. Over time, the body weight of mice in all groups gradually increased, with the MET, LDMY, and HDMY groups consistently showing lower body weights than the model group. Starting from the third week, the body weight of the LDMY and HDMY groups showed a decreasing trend (Figure 1B). These results indicate that DMY effectively regulates both blood glucose levels and body weight in *db/db* mice.

**Figure 2:**
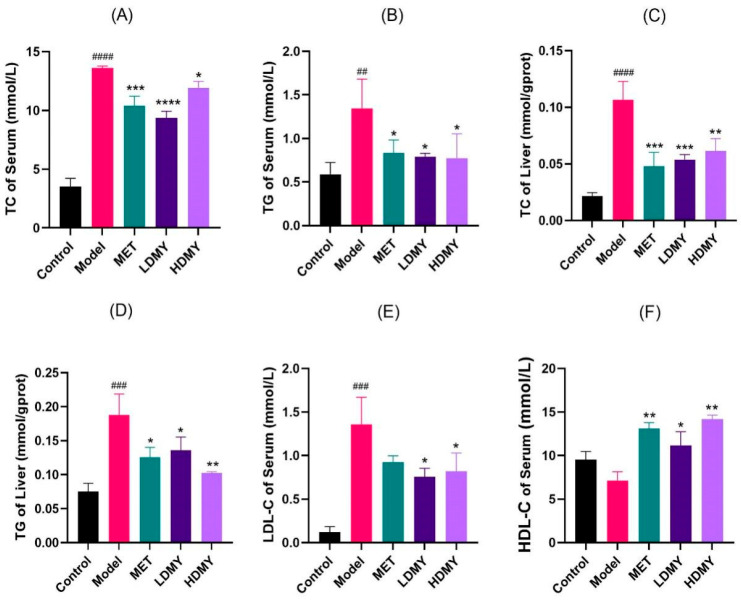
Effects of VDMY on serum TC (**A**), TG (**B**); liver TC (**C**), TG (**D**); serum LDL-C (**E**), HDL-C (**F**) in *db/db* mice. Control vs. model. ## *p* < 0.01, ### *p* < 0.001, #### *p* <0.0001. MET, LDMY, HDMY vs. model, * *p* < 0.05, ** *p* < 0.01, *** *p* < 0.001, **** *p* < 0.0001.

Regarding lipid metabolism, it was found that administration of VDMY remarkably reduced the levels of serum TC, TG, and LDL-C, and increased the level of serum HDL-C in *db/db* mice (*p* < 0.05). Moreover, liver TC and TG were found to exert a similar action as that in serum, with noteworthy differences noted (*p* < 0.05, Figure 2A–F). These results indicate that VDMY contributes to the reduction of blood lipid level in *db/db* mice, and VDMY has the capacity for liver chemoprevention.

### 2.2. Effects of VDMY on Liver Injury and Oxidative Stress in db/db Mice

**Figure 3:**
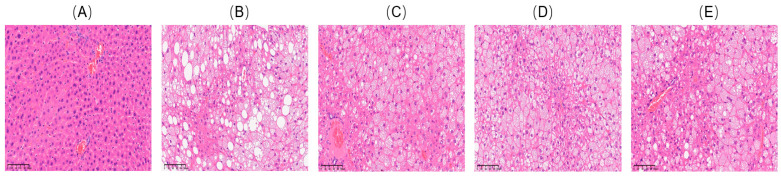
H&E staining of liver tissues of *db/db* mice: (**A**) control, (**B**) model, (**C**) MET, (**D**) LDMY, and (**E**) HDMY. The scale bar is 1:100 μm.

Subsequently, the effect of VDMY on liver chemoprevention and redox balance was investigated. To explore whether VDMY was able to mitigate liver injury, hematoxylin–eosin (H&E) staining of the liver was performed, and the results are shown in Figure 3. The liver tissue cells in the control exhibited a well-organized structure, characterized by typical cellular morphology, ample cytoplasmic content, and clearly defined boundaries (Figure 3A). In contrast, Figure 3B illustrates that the model displays a pronounced increase in the quantity of lipid droplets, a notable enlargement of the nuclei, and a diminution in the size of the liver lobules relative to the control. As depicted in Figure 3D,E, VDMY exhibited a notable ability to alleviate liver tissue damage in *db/db* mice that had been treated. This was evidenced by a substantial decline in the accumulation of liver fat droplets, a tendency towards the normalization of nuclear morphology, the maintenance of intact liver lobules, and a reduction in fatty degeneration. These findings reveal that VDMY may confer protective benefits for liver health and mitigate liver injury.

**Figure 4:**
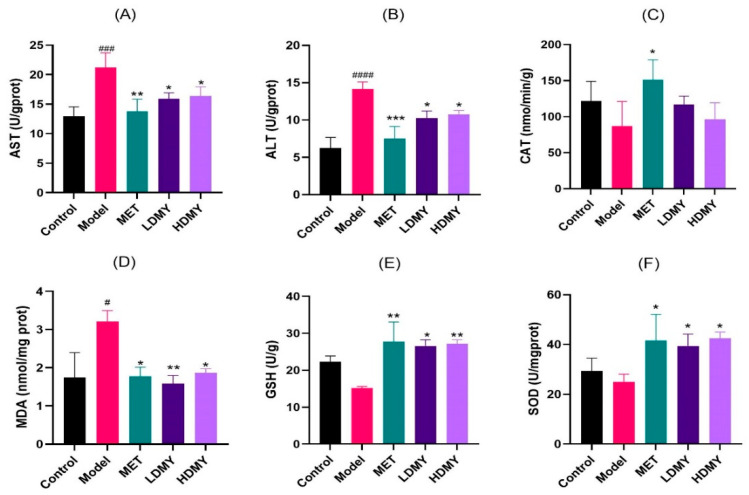
The effect of VDMY on AST (**A**), ALT (**B**), CAT (**C**), MDA (**D**), GSH (**E**), and SOD (**F**) levels in the liver tissues of *db/db* mice. Control vs. model. # *p* < 0.05, ### *p* < 0.001. #### *p* <0.0001. MET, LDMY, HDMY vs. model, * *p* < 0.05, ** *p* < 0.01, *** *p* < 0.001.

The impact of VDMY on liver oxidative stress was further assessed by measuring six biomarkers: AST, ALT, MDA, SOD, CAT, and GSH. As shown in Figure 4A–F, the model group notably increased the levels of AST, ALT, and MDA in liver tissues, compared to the control group (*p* < 0.05). Conversely, the levels of SOD, CAT, and GSH were markedly diminished in the model group. In contrast to the model group, the LDMY and HDMY groups exhibited a remarkable reduction in the levels of AST, ALT, and MDA (*p* < 0.05); while the levels of SOD and GSH were observably increased by VDMY treatment (*p* < 0.05). However, there was an observed increase in CAT level, but without statistical significance. These findings suggest that VDMY has the potential to ameliorate liver excess oxidative stress and recover redox balance in *db/db* mice.

**Figure 5:**
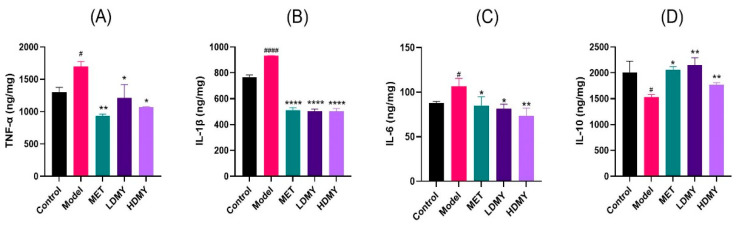
The effect of VDMY on TNF-α (**A**), IL-1β (**B**), IL-6 (**C**), and IL-10 (**D**) secretions in the liver tissues of *db/db* mice. Control vs. model, # *p* < 0.05, #### *p* < 0.0001. MET, LDMY, HDMY vs. model, * *p* < 0.05, ** *p* < 0.01, **** *p* < 0.0001.

It is important to note that the increased oxidative stress often triggers inflammatory responses; thus, several inflammatory factors were detected, such as TNF-α, IL-1β, IL-6, and IL-10. As illustrated in Figure 5A–D, the concentrations of pro-inflammatory cytokines TNF-α, IL-1β, and IL-6 were increased by DMY treatment, compared to the control group. In contrast, the concentration of the anti-inflammatory cytokine IL-10 exhibited a decrease, with statistically remarkable differences noted (*p* < 0.05). In comparison to the model group, the levels of pro-inflammatory factors TNF-α, IL-1β, and IL-6 in DMY groups all exhibited a decreasing trend, while the level of the anti-inflammatory factor IL-10 showed a significantly increasing trend (*p* < 0.05). These results suggest that VDMY has the potential to decrease liver endotoxin level in *db/db* mice, as well as to mitigate liver inflammatory responses.

### 2.3. Effects of VDMY on the Liver Transcriptome of db/db Mice

#### 2.3.1. Differential Gene Analysis

The gene data obtained by RNA-Seq were analyzed for differential expression, and the significantly different genes were screened under the condition of |log2FC| > 1 and *p* < 0.05. A pairwise comparison involving the four groups in relation to the model group is essential. This analysis will specifically entail comparisons between the model group and the control group, the model group and the MET group, the model group and the LDMY group, and the model group and the HDMY group. Gene identification, as a key component, aims to construct a comprehensive list of differentially expressed genes. Throughout the experimental data of Figure S1 in Supplementary Materials, the model group presented an impressive 3613 differentially expressed genes when compared to the control group, and when up- and down-regulated, the number of these genes amounted to 1687 and 1926, respectively. What is worth exploring in depth is that when the focus of the study shifted to the control analysis of the model group versus the MET group, the data revealed the presence of 3271 differentially expressed genes, of which up-regulated genes accounted for 1525, and down-regulated genes amounted to 1746. In the comparative study between the model group and the LDMY group, the experimental results demonstrated significant changes in 2095 differentially expressed genes, as evidenced by the fact that 1334 genes showed an up-regulation trend, while 761 genes exhibited down-regulation characteristics. Through an in-depth comparison of the model and HDMY groups, the study identified a total of 3103 differentially expressed genes, of which 1479 were up-regulated and 1624 were down-regulated.

#### 2.3.2. Differential Gene GO Enrichment Analysis

To explore the biological mechanism of VDMY during GLMD treatment, we used GO functional enrichment analysis to compare the gene expression differences between the LDMY and HDMY groups relative to the model group, with the aim of revealing the underlying functional regulatory networks. The Gene Ontology (GO) represents a comprehensive database that categorizes gene functions into three principal domains: biological process (BP), cellular component (CC), and molecular function (MF). The functional clustering within the GO framework utilizes a significance threshold of *p* < 0.05 to identify genes that were significantly enriched.

**Figure 6:**
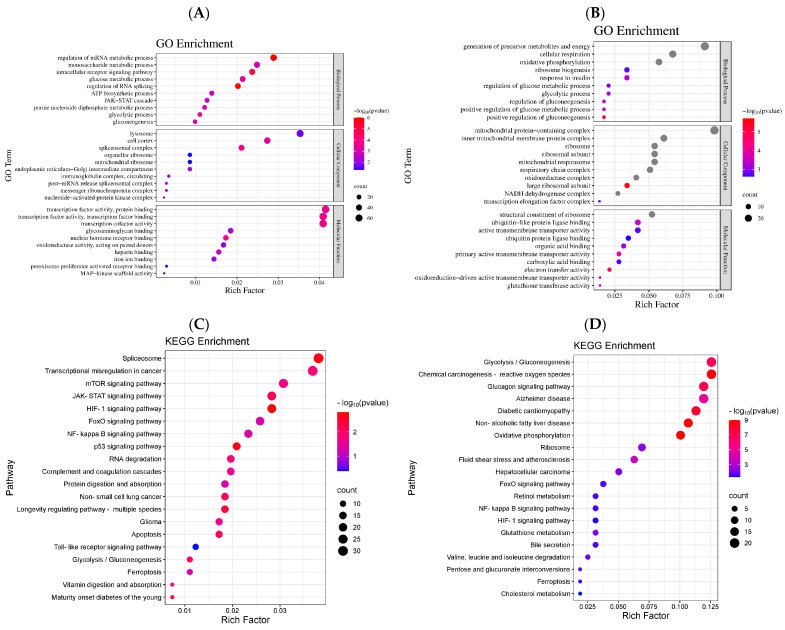
GO function analysis of differential gene expression. (**A**) Model vs. LDMY. (**B**) Model vs. HDMY. Differential gene expression was analyzed via the KEGG pathway. (**C**) Model vs. LDMY. (**D**) Model vs. HDMY.

By comparing the data of the model group with the data of the LDMY group, we unexpectedly found that the two showed strikingly enriched features in several biological categories. In biological processes, the most prominently enriched pathways included the regulation of transcription, glycolysis, gluconeogenesis, redox reactions, mRNA metabolic processes, and intracellular receptor signaling pathways. In cellular components, the key enriched entries were the spliceosome complex, cell cortex, endoplasmic reticulum-Golgi intermediate compartment, lysosome, mitochondrial ribosome, and messenger ribonucleoprotein complex. Regarding molecular functions, the enriched activities included transcription factor binding, protein binding, heparin binding, iron ion binding, oxidoreductase activity, and MAP kinase scaffold activity (Figure 6A).

As depicted in Figure 6B, the comparative analysis between the model group and the HDMY group indicates that the enriched categories within the biological process encompass the generation of precursor metabolites and energy, cellular respiration, oxidative phosphorylation, regulation of glucose metabolic processes, glycolytic processes, and positive regulation of gluconeogenesis, among other related entries. In the context of the cellular component, the enriched entries consist of the mitochondrial protein-containing complex, inner mitochondrial membrane protein complex, mitochondrial respirasome, ribosomal subunit, and respiratory chain complex, in addition to various other mitochondrial and ribosomal structures. Regarding molecular function, the primary enriched terms include structural constituents of the ribosome, active transmembrane transporter activity, electron transfer activity, carboxylic acid binding, and other pertinent molecular functions.

The results of the Gene Ontology (GO) enrichment analysis indicate that VDMY improves lipid metabolism irregularities in *db/db* mice through the modulation of several biological processes, such as transcription, glycolysis, gluconeogenesis, and redox reactions.

#### 2.3.3. Differential Gene KEGG Pathway Enrichment Analysis

Based on the findings from the GO enrichment analysis, the KEGG database was utilized to perform functional annotation and pathway enrichment analysis of the differentially expressed genes in the model vs. LDMY and model vs. HDMY groups. These analyses provided additional insights into the biological functions and signaling pathways involved, as shown in Figure 6C,D. As illustrated in Figure 6C, the comparison between the model group and the LDMY group revealed enrichment in several pathways, including the HIF-1 signaling pathway, p53 signaling pathway, Maturity Onset Diabetes of the Young, JAK-STAT signaling pathway, apoptosis, glycolysis/gluconeogenesis, RNA degradation, mTOR signaling pathway, Toll-like receptor signaling pathway, ferroptosis, FOXO signaling pathway, and NF-κB signaling pathway, among others.

Similarly, Figure 6D presents the enriched pathways identified in the comparison between the model group and the HDMY group. Key pathways include chemical carcinogenesis—reactive oxygen species, non-alcoholic fatty liver disease, glucagon signaling pathway, glycolysis/gluconeogenesis, metabolic interconversions, FOXO signaling pathway, HIF-1 signaling pathway, cholesterol metabolism, NF-κB signaling pathway, and ferroptosis, among others.

The results of the KEGG pathway enrichment analysis indicated that the Toll-like receptor signaling pathway, NF-κB signaling pathway, and FOXO signaling pathway were selected for validation. These pathways were identified as enriched signaling pathways connected with differentially expressed genes related to abnormal glucose and lipid metabolism in *db/db* mice subjected to VDMY intervention.

### 2.4. Effects of VDMY on the TLR4/MyD88/NF-κB and FOXO1 Signaling Pathways in db/db Mice Liver

**Figure 7:**
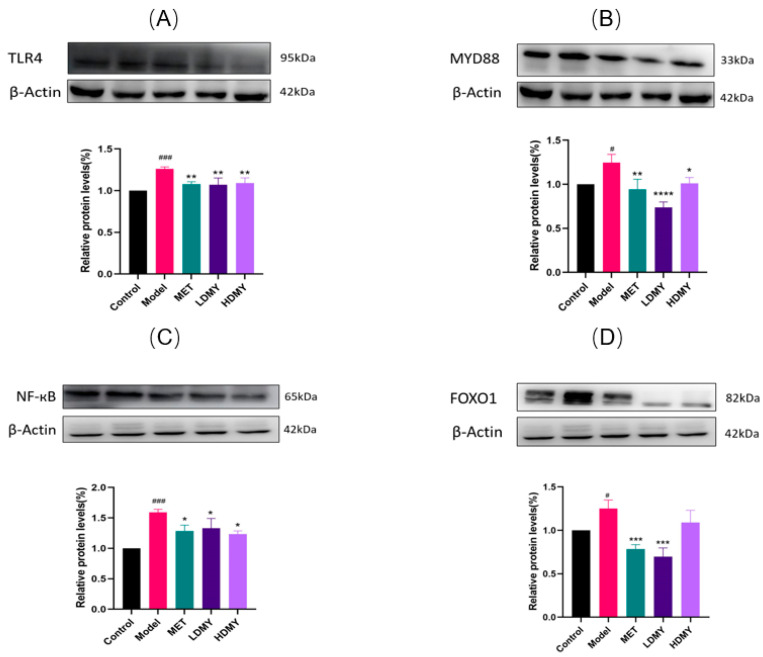
The effect of VDMY on TLR4 (**A**), MyD88 (**B**), NF-κB (**C**), and FOXO1 (**D**) expressions in the liver tissues of *db/db* mice. Control vs. model. # *p* < 0.05, ### *p* < 0.001. MET, LDMY, HDMY vs. model, * *p* < 0.05, ** *p* < 0.01, *** *p* < 0.001, ****p < 0.0001.

Through the in-depth analysis of KEGG pathway enrichment data, we unexpectedly discovered two signaling pathways of great research value—Toll-like receptor (TLR4) signaling pathway and FOXO1 signaling pathway, which play an indispensable role in regulating metabolic homeostasis and inflammatory response in *db/db* mice. Consistent with the KEGG findings, Western blot analysis (Figure 7) demonstrated that, compared to the control group, the expression levels of TLR4, MyD88, NF-κB, and FOXO1 proteins were significantly elevated in the livers of *db/db* mice in the experimental group (*p* < 0.05). The experimental data were validated by Western blotting (Figure 7), and the data coincided with the pathway regulation pattern revealed by KEGG analysis. It is worth noting that the expression of key proteins, including TLR4, MyD88, NF-κB, and FOXO1, was significantly up-regulated in the liver tissues of *db/db* mice in the experimental group compared with that of the control group (*p* < 0.05), which not only confirms our theoretical hypothesis but also provides a strong support for an in-depth understanding of the molecular mechanism of metabolic disorders. These findings imply that the protective mechanism of VDMY in *db/db* mice is mediated through the suppression of NF-κB activation via the TLR4/MyD88 signaling pathway and FOXO1. This mechanism aligns with the pathway-level insights revealed by the KEGG analysis, thereby further underscoring the critical role of the TLR4 signaling axis in the therapeutic effects of VDMY.

### 2.5. Correlation Analysis

**Figure 8:**
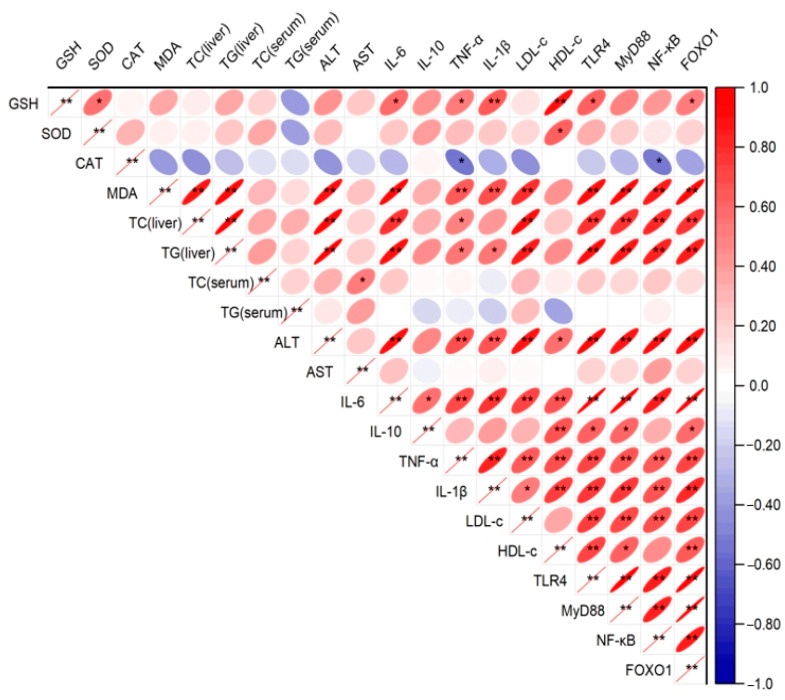
Correlation heatmap of biochemical parameters. * *p* < 0.05; ** *p* < 0.01.

The correlation matrix (Figure 8) illustrates the relationships among oxidative stress markers, inflammatory cytokines, metabolic indicators, and signaling proteins in the liver and serum of *db/db* mice. Notably, TLR4, MyD88, NF-κB, and FOXO1 exhibited strong positive correlations, reflecting their coordinated roles within the TLR4/MyD88/NF-κB signaling pathway and FOXO1-associated regulatory mechanisms. The oxidative stress marker MDA demonstrated a strong negative correlation with antioxidant indicators such as GSH, SOD, and CAT, highlighting the inverse relationship between oxidative stress and antioxidant defenses. Furthermore, inflammatory cytokines, including IL-6, TNF-α, and IL-1β, displayed significant positive correlations with NF-κB, reinforcing its central role in mediating inflammatory responses. Metabolic markers, such as TG and TC, were positively correlated with pro-inflammatory cytokines and NF-κB, suggesting an interplay between metabolic dysregulation and inflammation. Conversely, HDL-c was negatively correlated with NF-κB and inflammatory markers, indicating its potential anti-inflammatory effect. These findings collectively underscore the pivotal roles of TLR4/MyD88/NF-κB signaling, FOXO1, and oxidative stress in the pathophysiology of *db/db* mice and provide insights into potential therapeutic targets.

## 3. Discussion

In recent years, natural plant extracts have attracted considerable attention owing to their notable effectiveness in the management of metabolic disorders associated with glucose regulation, alongside a low incidence of adverse effects. These extracts possess the potential to address the diverse and complex health needs of the population. DMY is the primary constituent of vine tea extract and represents a significant bioactive compound within the flavonoid class. DMY exhibits a diverse array of physiological activities and demonstrates a favorable safety profile. Furthermore, advancements in formulation technologies have significantly enhanced its bioavailability. In recent years, investigations into the effects of DMY on glucose and lipid metabolism disorders have primarily concentrated on specific signaling pathways associated with oxidative stress and inflammation. Nevertheless, the mechanisms through which DMY exerts its effects have not been comprehensively and systematically explored [33,34,35]. In the present investigation, we established that VDMY significantly decreases blood glucose and lipid concentrations in *db/db* mice. Additionally, VDMY was found to markedly enhance liver oxidative-stress parameters and reduce hepatic injury. Our results further suggest that VDMY mitigates liver damage and inflammation by inhibiting the TLR4/MyD88/NF-κB signaling pathway.

GLMD is typically characterized by significant elevations in blood glucose levels, TG, TC, and LDL-C, accompanied by a reduction in HDL-C [36]. These metabolic abnormalities contribute to oxidative stress, defined as a disruption in the balance between oxidants and antioxidants. This imbalance leads to the excessive accumulation of oxidants, which can result in cellular and tissue damage. SOD, MDA, CAT, and GSH are critical biomarkers and enzymes involved in oxidative stress regulation, collectively maintaining intracellular redox equilibrium and serving as reliable indicators of oxidative stress [37,38]. The liver, as the primary metabolic organ, plays a pivotal role in the metabolism and detoxification of endogenous and exogenous substances. During hepatic injury, ALT, AST, and MDA levels are significantly elevated, while the activities of SOD, CAT, and GSH are markedly reduced [39]. In this study, VDMY was shown to exert a notable hypoglycemic effect, significantly reducing TG, TC, and LDL-C levels while increasing HDL-C levels, consistent with the effects observed in the MET-treated group. In the context of liver injury and oxidative stress, VDMY significantly decreased ALT, AST, and MDA levels while enhancing the activities of CAT, GSH, and SOD. These results align with the findings of Bin Wu et al., who reported that DMY alleviates oxidative stress in diabetic mice by reducing MDA levels and increasing SOD and GSH activities [40]. Similarly, Hongyan Ling et al. demonstrated that DMY ameliorates dyslipidemia abnormalities associated with diabetes by decreasing TG, TC, and LDL-C levels while increasing HDL-C levels [24]. Collectively, these findings corroborate our results and highlight the potential of VDMY in mitigating oxidative stress, hepatic injury, and glucose–lipid metabolism disorders.

Inflammation plays an essential role in the pathogenesis of metabolic disorders. In this complex molecular regulatory network, pro-inflammatory factors such as TNF-α, IL-1β, and IL-6 continuously erode the integrity of insulin signaling through multiple signaling cascades, ultimately reducing the biological effects of insulin in the cell, and triggering a series of pathological changes centered on insulin resistance. Lianjie Hou and associates identified that DMY has the capacity to decrease the concentrations of TNF-α, IL-1β, and IL-6 in diabetic mice that were placed on a high-fat diet [41]. Guan et al. [42] demonstrated that DMY possesses the ability to reduce the concentrations of TNF-α and IL-1β, consequently mitigating inflammation related to diabetes. In this study, we observed that, following the administration of VDMY, there was a significant downregulation in the levels of pro-inflammatory factors TNF-α, IL-1β, and IL-6, while the level of the anti-inflammatory factor IL-10 exhibited a noteworthy upregulation. These results align with the findings presented in the previously referenced paper.

In studying the mechanism of DMY, Zhou et al. [43] conducted a study examining the impact of DMY on insulin resistance, revealing that it improves insulin sensitivity by upregulating AMPK, PGC-1α, p-Akt, and p-IRS-1. Furthermore, additional studies have suggested that DMY may alleviate type 2 diabetes by inhibiting the phosphorylation of PPARγ and ERK [33,44]. A primary objective of RNA-seq profiling is to identify genes or molecular pathways that exhibit differential expression (DE) across multiple biological conditions [45]. In this research, we determined that VDMY modulates key transcriptional mechanisms and metabolic pathways, including glycolysis, gluconeogenesis, and redox reactions, highlighting its regulatory potential in glucolipid metabolism and an annotation of the TLR4/MyD88/NF-κB signaling pathway through the application of RNA sequencing (RNA-seq) techniques. Toll-like receptors (TLRs) represent a prominent group of pattern-recognition receptors (PRRs) that play a crucial role in the activation of the NF-κB inflammatory signaling pathway [46]. Myeloid differentiation primary response 88 (MyD88) serves as a crucial adaptor protein for all Toll-like receptors (TLRs). Upon the recognition of its ligand by TLR4, MyD88 facilitates the transmission of signals. MyD88 contains a Toll/interleukin-1 receptor (TIR) domain that interacts with the TIR domain of TLR4, thereby initiating the process of signal transduction [47]. Upon activation of the TLR4/MyD88 signaling pathway, MyD88 initiates the activation of downstream kinases, resulting in the translocation of the NF-κB transcription factor into the cell nucleus. This translocation enables the transcription of genes linked to inflammatory processes. The TLR4/MyD88/NF-κB pathway assumes a pivotal function in controlling the expression of numerous genes tied to inflammation, driving the onset and regulation of the organism’s inflammatory response. Through precise molecular interactions, this pathway orchestrates a cascade of signaling events, effectively bridging extracellular stimuli with intracellular transcriptional activity, and thereby fine-tuning the dynamic balance of pro-inflammatory and anti-inflammatory mediators. This study aims to investigate the potential mechanisms through which VDMY exerts a protective effect against inflammation and liver injury by modulating the TLR4-mediated NF-κB signaling pathway. We systematically evaluated the expression levels of key proteins in the TLR4/MyD88/NF-κB signaling pathway through in-depth exploration of the Western blotting technique. The experimental data encouragingly revealed an important finding: VDMY exhibited a significant inhibitory effect in the liver tissues of *db/db* mice, effectively suppressing the over-activation of the TLR4/MyD88/NF-κB signaling pathway. This finding not only provides a new perspective for us to understand the mechanism of VDMY, but it also suggests that VDMY has a promising application in alleviating diabetes-induced liver injury and inflammation.

This study acknowledges certain limitations. The investigation focused on the effects of VDMY on the intestinal barrier, gut microbiota, intestinal inflammation, and bile acid metabolism. We systematically analyzed the molecular mechanisms by which VDMY influences glucose and lipid metabolism through the gut–liver axis. In conclusion, this research provides innovative perspectives on the prevention and management of liver inflammation and metabolic diseases linked to glucose and lipid metabolic disorders, achieved through the application of VDMY.

## 4. Materials and Methods

### 4.1. Materials and Reagents

The vine tea extract was procured from Changsha Green Vine Biotechnology Co., Ltd. in Changsha, China, yielding a dihydromyricetin (VDMY, CAS: 27200-12-0/529-44-2) powder with a purity of 65% through a water extraction process (Figures S2 and S3). Metformin hydrochloride (MET), with a minimum purity of 99%, was acquired from Beijing Jing Feng Pharmaceutical Group Co., Ltd., in Beijing, China.

### 4.2. Animal Models

A total of forty male *db/db* mice and ten C57BL/6 mice, aged 8 weeks and maintained in a specific pathogen-free environment, were housed in a barrier facility at the Hunan Animal Experimental Center (HNSE2024(5)023). SPF-grade male *db/db* mice were fasted for 12 h, and fasting blood glucose was measured. After 1 week of acclimatization, 10 C57BL/6 mice served as the control group. A total of 40 male *db/db* mice, characterized by fasting blood glucose levels exceeding 11.1 mmol/L and weighing between 28.9 g and 36.0 g, were randomly divided into four groups: model, MET (MET, 0.26 g/kg), LDMY (low-dose VDMY, 150 mg/kg), and HDMY (high-dose VDMY, 300 mg/kg), with 10 mice assigned to each group. Each treatment group received the corresponding drug solution via gavage once daily for a period of 28 consecutive days. The body weight and blood glucose levels were assessed weekly. On the 28th day, organ samples were collected from each group and subsequently frozen at −80 °C.

### 4.3. Serum and Liver Biochemical Indicators

Biweekly, blood samples were obtained from the retro-orbital venous plexus of each cohort of mice and were then subjected to centrifugation at 3000 rpm for a duration of 10 min to separate the serum. With the special test kit from Nanjing Institute of Biotechnology (Nanjing, China), we were able to explore the specific levels of various lipid indicators in serum, including key biochemical parameters such as LDL-C, total cholesterol (TC), TG, and HDL-C. The supernatant was collected, and the assay kit provided by Nanjing Biotechnology Research Institute was utilized to quantify the levels of SOD, reduced glutathione peroxidase (GSH), aspartate aminotransferase (AST), and alanine aminotransferase (ALT). Furthermore, the assay kit from Suzhou Comin bio (Suzhou, China) was employed to measure the concentration of CAT. In this study, a high-precision enzyme-linked immunosorbent assay (ELISA) kit developed by Shanghai Heng yuan Biotechnology Co., Ltd. (Shanghai, China), was used to quantitatively analyze the expression levels of key inflammatory factors, such as TNF-α, IL-10, IL-6, and IL-1β. The whole process of the test was carried out in strict accordance with the operating procedures attached to the kit, which not only ensured the accuracy of the data but also laid a solid foundation for the subsequent research.

### 4.4. Organizational Pathology Analysis

At the end of the fourth week of the experiment, each group of mice was deeply anesthetized, and then blood samples were taken through the abdominal aorta, followed by humane euthanasia. The left lobe of each rodent’s liver was precisely located, and tissue samples were taken from specific areas and immersed in a 4% paraformaldehyde solution for storage for further study. Following a 24-h incubation period, the liver samples were embedded in paraffin and subsequently sectioned with a low-temperature microtome. The resultant sections were stained with hematoxylin–eosin (H&E) to enable histological examination. Ultimately, pathological imaging was performed using the DFC420C pathology imaging system (Leica, Wetzlar, Germany).

### 4.5. Liver RNA Sequencing

The extraction and detection of total RNA from mice in each experimental group were performed by Beijing NoVo Gene Technology Co., Ltd. (Beijing, China). RNA isolation and purification from samples were achieved with TRIzol. Library construction started with total RNA extraction, followed by poly(A) mRNA selection with Oligo(dT) beads. Subsequently, the mRNA underwent targeted fragmentation through the introduction of a fragmentation buffer containing divalent cations, effectively inducing random cleavage at specific sites along the mRNA strand. The synthesis of the initial strand of complementary DNA (cDNA) was conducted utilizing fragmented mRNA as a template and random oligonucleotides as primers within the M-MuLV reverse-transcriptase system. Ribonuclease H degrades RNA, and DNA polymerase I builds cDNA using dNTPs, forming a complex yet elegant molecular process. Through end repair, tail clipping, and ligation, double-stranded cDNA was amplified by PCR. To maintain library purity, AMPure XP beads were used for efficient purification, crucial for subsequent studies. Preliminary quantification of the library was performed with the Qubit2.0 Fluorometer (Life Technologies, Carlsbad, CA, USA), followed by the Agilent 5400 bioanalyzer (Agilent Technologies, Colorado Springs, CO, USA) qRT-PCR confirmed the library’s effective concentration and quality. After qualified library inspection, Illumina sequencing was performed, and end readings were generated.

### 4.6. GO Enrichment and KEGG Pathway Analysis

Gene Ontology (GO) enrichment analysis of differentially expressed genes was implemented by the cluster Profiler R package (Version 3.5.0) in which gene-length bias was corrected. GO terms with corrected *p*-values less than 0.05 were considered significantly enriched by differential expressed genes. KEGG is a database resource for understanding high-level functions and utilities of the biological system (http://www.genome.jp/kegg/, accessed on 25 February 2025). We used cluster Profiler R package to test the statistical enrichment of differential expression genes in KEGG pathways.

### 4.7. Western Blotting

We extracted liver tissue protein using Beijing Solarbio’s kit (Beijing, China), following the instructions; centrifuged at 8000× *g* for 10 min at 4 °C; and then collected proteins. Following this, the supernatant should be collected, and the protein concentration should be evaluated using the BCA protein assay kit (Beyotime Biotech Inc., Shanghai, China). The proteins are to be separated utilizing 10% SDS-PAGE, after which they should be transferred to a PVDF membrane (Amersham Pharmacia Biotech, Amersham, UK). The membrane was blocked for 1 h at room temperature. Following this step, the membrane should be incubated with the primary antibody overnight at a temperature of 4 °C. The primary antibodies employed for TLR4, MyD88, NF-κB, and FOXO1 were obtained from Cell Signaling Technology (CST, Waltham, MA, USA) and were diluted at a ratio of 1:2000. The anti-β-Actin was acquired from Proteintech Group, Inc., Wuhan, China and was used at a dilution of 1:20,000. Afterward, the membrane should be incubated with the enzyme-labeled secondary antibody for an additional duration of one hour. The secondary antibody employed in this research was sourced from an anti-rabbit antibody (Proteintech Group, Inc., Wuhan, China), and it was diluted to a concentration of 1:20,000. A sufficient quantity of ECL chemiluminescent solution was subsequently administered to the PVDF membrane, which was then exposed and developed using the Image Quant LAS 4000 mini (General Electric Company, Morrison, CO, USA). The quantification of the protein bands was conducted utilizing software ImageJ (1.53t, https://imagej.net/ij/, accessed on 25 February 2025).

### 4.8. Correlation Analysis

All the original data of the experiment were put into Origin 2024b for correlation analysis.

### 4.9. Statistical Analysis

Each experiment was executed in triplicate, with SPSS 26.0 employed for the analysis of individual trial outcomes. Graphical representations of the data were generated with GraphPad Prism version 9. The data were expressed as the mean ± SD. Statistical significance was assessed using one-way ANOVA with multiple comparisons, with a *p-*value of less than 0.05 considered to be statistically important.

## 5. Conclusions

In conclusion, this study demonstrates that the water extract of vine tea (VDMY) significantly reduces blood glucose and lipid levels in *db/db* mice, effectively mitigating oxidative stress-induced hepatic damage. Furthermore, VDMY modulates key transcriptional mechanisms and metabolic pathways, including glycolysis, gluconeogenesis, and redox reactions, highlighting its regulatory potential in glucolipid metabolism. Notably, VDMY exhibits a profound inhibitory effect on the TLR4/MyD88/NF-κB and FOXO1 signaling pathways, contributing to reduced hepatic injury and inflammation. These findings provide a novel theoretical foundation for the application of VDMY as a targeted dietary intervention in precision nutrition, offering promising therapeutic strategies for managing glucose and lipid metabolism disorders.

Undoubtedly, this study has certain limitations. In the future, we will explore the effects of VDMY on gut microbiota and bile acid metabolism, among other aspects, and further investigate the molecular mechanisms through which VDMY regulates glucolipid metabolism disorders. This will contribute to positioning VDMY as a potential natural dietary intervention for glucolipid metabolism disorders.

## Data Availability

All data generated or analyzed during this study are available from the corresponding author upon reasonable request.

## Supplementary Materials

The following supporting information can be downloaded at: https://www.mdpi.com/article/10.3390/ijms26052169/s1.
