# Supplementary material for: Molecular Mechanism of Vine Tea Dihydromyricetin Extract on Alleviating Glucolipid Metabolism Disorder in db/db Mice: Based on Liver RNA-Seq and TLR4/MyD88/NF-κB Pathway
Source: Int J Mol Sci. 2025 Feb 28;26(5):2169. doi: 10.3390/ijms26052169 (PMC11900051; doi:10.3390/ijms26052169)
Supplement: Supplementary file 1 [file ijms-26-02169-s001.zip › ijms-3466154-supplementary.pdf]

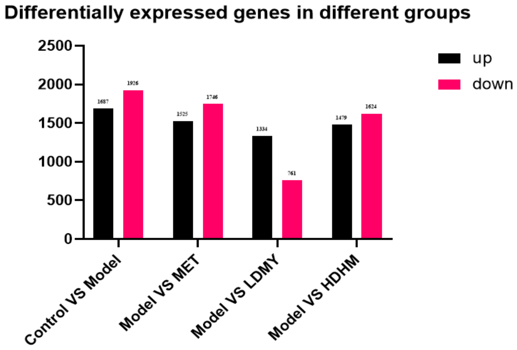

Figure S1. Differential gene expressions between each group.

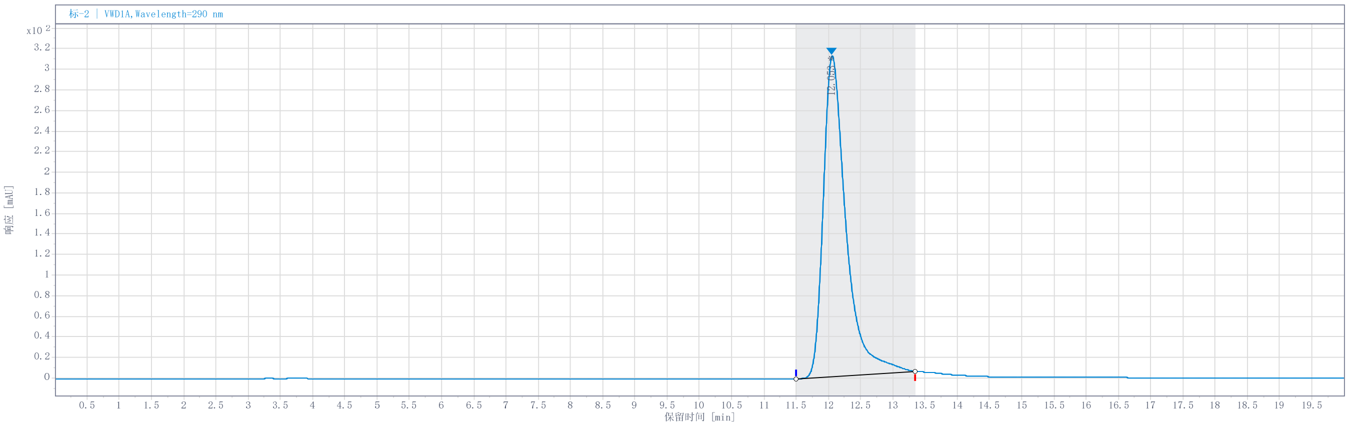

Figure S2. Control：99.26% 18.24mg—50ml of DMY HPLC

#	RT (min)	stop time (min)	Peak area	Peak area %
1	12.053	13.338	7681.478	100.000

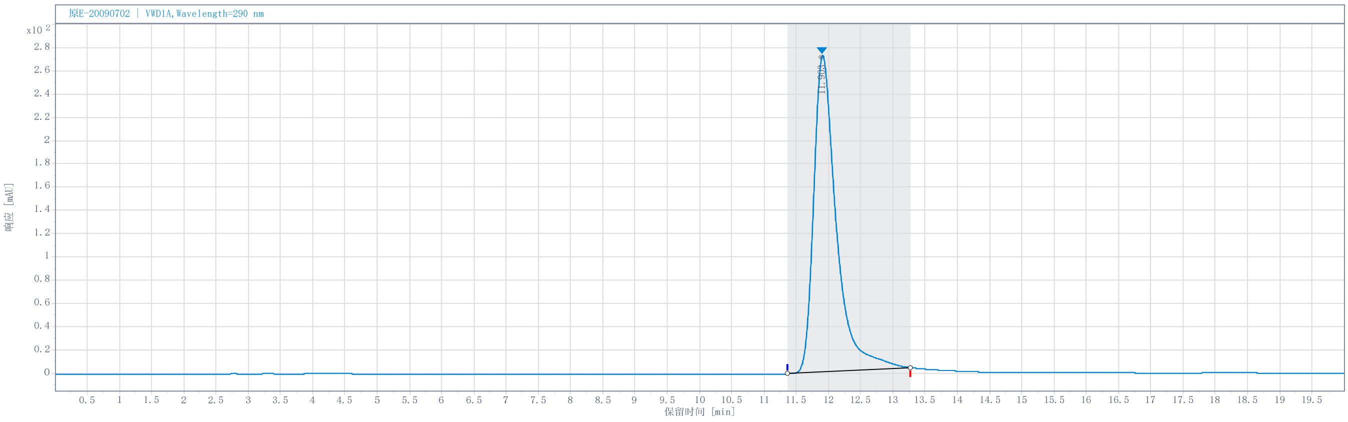

Figure S3. Sample：11.86mg—25ml 65.75% of VDMY HPLC

#	RT (min)	stop time (min)	Peak area	Peak area %
1	11.903	13.278	6684.361	100.000
